# miR-185 and SEPT5 Genes May Contribute to Parkinson's Disease Pathophysiology

**DOI:** 10.1155/2019/5019815

**Published:** 2019-11-14

**Authors:** Arman Rahimmi, Ilaria Peluso, Aref Rajabi, Kambiz Hassanzadeh

**Affiliations:** ^1^Student Research Committee, Kurdistan University of Medical Sciences, Sanandaj, Iran; ^2^Cellular and Molecular Research Center, Research Institute for Health Development, Kurdistan University of Medical Sciences, Sanandaj, Iran; ^3^Council for Agricultural Research and Economics, Research Centre for Food and Nutrition (CREA-AN), Via Ardeatina 546, 00178 Rome, Italy; ^4^Department of Neurology, Kurdistan University of Medical Sciences, Sanandaj, Iran; ^5^Department of Physiology and Pharmacology, Kurdistan University of Medical Sciences, Sanandaj, Iran

## Abstract

There are still unknown mechanisms involved in the development of Parkinson's disease (PD), which elucidating them can assist in developing efficient therapies. Recently, studies showed that genes located on the human chromosomal location 22q11.2 might be involved in the development of PD. Therefore, the present study was designed to evaluate the role of two genes located on the chromosomal location (miR-185 and SEPT5), which were the most probable candidates based on our bibliography. *In vivo* and *in vitro* models of PD were developed using male Wistar rats and SHSY-5Y cell line, respectively. The expression levels of miR-185, SEPT5, LRRK2, and PARK2 genes were measured at a mRNA level in dopaminergic areas of rats' brains and SHSY-5Y cells using the *SYBR Green Real-Time PCR Method*. Additionally, the effect of inhibition on the genes or their products on cell viability and gene expression pattern in SHSY-5Y cells was investigated. The level of miR-185 gene expression was significantly decreased in the substantia nigra (SN) and striatum (ST) of the rotenone-treated group (control group) compared to the healthy normal group (*P* < 0.05). In addition, there was a significant difference in the expression of SEPT5 gene (*P* < 0.05) in the substantia nigra between two studied groups. The results of an *in vitro* study showed no significant change in the expression of the genes; however, the inhibition on miR-185 gene expression led to the increase in LRRK2 gene expression in SHSY-5Y cells. The inhibition on LRRK2 protein also decreased the cellular toxicity effect of rotenone on SHSY-5Y cells. The results suggested the protective role of miR-185 gene in preventing the development of PD.

## 1. Introduction

Parkinson's disease (PD) is a prevalent central nervous system (CNS) disorder, which develops due to the loss of nigrostriatal dopaminergic neurons in the midbrain [1]. Whereupon, movement (resting tremor, muscular rigidity, bradykinesia, gait impairment, etc.) and behavioral (cognitive impairment and dementia) disorders appeared [2]. The prevalence of PD is about 1-2% in people older than 65 years old, 4% in people older than 85 years old, and 0.3% in total population [3]. About one million people suffer from PD in United States of America and 50,000 to 60,000 new cases are diagnosed every year. The prevalence of PD is estimated to increase two folds until 2030 [4]. The economic burden of PD is estimated to be 10.8 billion dollars annually only in the United States; out of which, 58% is directly related to the medical care services [4]. Furthermore, PD is a chronic and progressive disease, lasting for years and decreasing life quality of the patients intensely [5]. Thus, finding factors causing PD has a great importance, since it can help uncovering the nature of the disease and developing more efficient therapies.

Researchers have determined many factors for the development of PD, including environmental toxins (e.g., pesticides and herbicides), genetic background (mutations in particular genes such as SNCA, PINK1, Parkin, LRRK2, and DJ-1), certain viruses and/or a combination of all [6, 7]. Importantly, most of the recent studies show that no matter what the cause is, oxidative stress and inflammation always play a key role in the death of the dopaminergic cells of substantia nigra (SN) [8, 9]. Oxidative stress is caused by many external and internal sources, such as certain toxins and pathogens, dopamine metabolism, mitochondrial impairment, and reactive iron stored in neuromelanin [10]. Numerous studies frequently reported the increase in the inflammatory factors, such as TNF-*α*, NF-*κ*B, IL-6, and IL-2 in the SN of PD patients and PD animal models [11, 12]. Indubitably, PD is a multifactorial disease, which many factors can contribute in its development, either alone or accompanied with other factors, although neither all causing factors are discovered yet nor complete molecular mechanism of the disease is known.

Recently, researchers discovered a locus on human chromosome 22 (22q11.1), which its congenital absence due to the microdeletion (DiGeorge syndrome) could lead to the early onset of PD [13]. The locus is consisted of about 30 genes; none of them are among the genes known to be involved in the development of PD [13, 14]. Interestingly, it appears that some of the genes in 22q11.2 locus are probable candidates involving in the molecular mechanisms of the development and progression of PD. miR-185 is one of those genes predicted to target LRRK2 [15, 16]. SEPT5 is another gene located on 22q11.2 locus, which its protein product interacts with the products of certain genes such as PARK2 and catechol-O-methyltransferase (COMT) [14]. LRRK2 is a kinase present in cytoplasm and outer membrane of mitochondria. Mutations or overexpressions of LRRK2 gene are strongly associated with development of Parkinson's disease [17]. PARK2 is a ligase which assist proteasome complexes to degrade misfolded proteins. Mutations or overexpressions of PARK2 gene are also strongly associated with development of Parkinson's disease [18].

On the other hand, some researchers believe that disorders such as epilepsy, schizophrenia, and failure of certain internal secretory glands (thyroid, parathyroid, and thymus) are more straightforward consequences of 22q11.2 microdeletion. They suggest that PD is caused as a result of the psychological drugs, consumed by the patients with DiGeorge syndrome [19]. However, more recent studies challenge this belief, since it is observed that the patients with DiGeorge syndrome show movement disorders even in their childhood. Additionally, PD develops in those adult patients with DiGeorge syndrome, who are not under the treatment with psychological drug regimens [13, 20]. Therefore, PD can be considered to be an independent aspect of 22q11.2 microdeletion, not a result of psychological drug consumption.

Despite the potential role of those two genes (miR-185 and SEPT5), only a few studies showed the possible role of the genes in the development of PD. Therefore, evaluation regarding the expression of these genes can provide a new and important insight into the pathogenesis of PD.

In order to investigate the possible role of miR-185 and SEPT5 genes in the pathogenesis of PD, their transcript expression level was assessed in SHSY-5Y cell line (a human cell line) treated by rotenone (*in vitro* model of PD) and brain tissues (substantia nigra and striatum) of male Wistar rat as the *in vivo* model of PD (induced by rotenone). The expression of two molecular targets of miR-185 and SEPT5 (i.e., LRRK2 and PARK2) was assessed to determine whether there is a presumptive relation among the two genes (miR-185 and SEPT5) and the known genes causing PD.

## 2. Materials and Methods

### 2.1. Chemicals

Rotenone, sunflower oil and DMSO were purchased from Sigma-Aldrich Company (St. Louis, MO, USA). HG-10-102-01 (LRRK2 inhibitor) was purchased from Cayman Chemical Company (New York, NY, USA). miRZip-185 was purchased from Sanbio Company (Uden, Netherlands).

### 2.2. Animals

Sixteen male Wistar rats (Pasteur Institute of Iran) weighing 300 ± 25 g and aged 5 months were selected randomly. Animals were housed in polycarbonate cages (2 rats per cage) under controlled conditions, including 12 h light/dark cycles, the environment temperature of 23 ± 2°C, and the environment humidity of 60 ± 5%. Standard food and water were accessible for the animals ad libitum. All experiments were performed, while considering the Guide for the Care and Use of Laboratory Animals (National Institutes of Health Publication No. 85-23, revised 1985). The research ethics committee of Kurdistan University of Medical Sciences also approved all experiments in this study.

### 2.3. Experimental Groups

Animals were randomly divided into two groups (*n* = 8), which are as follows:

- The control group consisting of the rats received rotenone (1.5 mg/kg/24 h, SC)
- The healthy normal group consisting of the rats received rotenone vehicle (1 ml/kg/24 h, SC)

The sample size was calculated by G power software.

### 2.4. Method Used for Induction of Parkinson's Disease in Animals

Rotenone solution was freshly prepared before injections. So that, it was dissolved in dimethyl sulfoxide. Then, sunflower oil was added to the solution to dilute it and reach a final concentration of 1.5 mg/ml of rotenone. The ratio of dimethyl sulfoxide to sunflower oil in the final solution was equal to 2 : 98. In order to induce Parkinson's disease, animals received rotenone at a concentration of 1.5 mg/kg each for 24 h. The injections continued until motor dysfunction was declared on all behavioral tests (rotarod test, bar test, and rearing test) in the control group. Animals were tested every 5 days, before daily injection of rotenone. The day in which the significant decrease was observed in all behavioral tests was considered as the day of model development of Parkinson's disease (46^th^ day since the first injection). On the last day, all the behavioral tests were repeated 10 min after injection of apomorphine (1 mg/kg, SC), to confirm whether the motor dysfunctions were dopamine-dependent and not a result of the unspecific consequences of rotenone treatment on peripheral organs. This protocol was a modified protocol based on our previous studies and was conducted several times as pilot, in order to be optimized in our lab [21]. The methods used for performing behavioral tests are presented in details in one of our previous studies [22].

### 2.5. Body Weight Measurement

Animals were weighted every 48 h before the injections, and it was done to be an index for monitoring the health condition and possible peripheral toxicity induced by rotenone. Indeed, a significant decrease in the body weight shows the unspecific effects of rotenone treatment [23].

### 2.6. Tissue Collection

At the end of the motor and behavioral assessments (46^th^ day), the animals were sacrificed by decapitation under deep anesthesia using ketamine/xylazine cocktail. The substantia nigra and striatum were removed, cleaned, and frozen in liquid nitrogen and then were stored at −80°C until use.

### 2.7. Extraction of Total RNA, cDNA Synthesis, and Real-Time PCR

Total RNA was extracted from the frozen tissues of animals or isolated cells obtained from cell cultures within 2 weeks after collecting samples, using the total RNA extraction kit (Favorgen, Thailand). Briefly, approximately 50 mg of frozen brain tissue was homogenized by an ultrasound homogenizer on ice in a cold anti-RNase-containing buffer, and extraction processing was carried out according to the manufacturer's instructions. RNA concentration and purity was determined by the absorbance at 230, 260, and 280 nm.

Reverse transcription was conducted by the cDNA synthesis kit (Favorgen, Thailand). Briefly, 1 *μ*g of RNA, 1 *μ*l of random hexamer primer, and appropriate amount of DEPC-treated water up to 13.5 *μ*l were mixed in a 0.2 ml microtube. Then, the mixture was incubated at 70°C for 5 min and was chilled on ice. Next, 4 *μ*l of 5x first-strand buffer, 1 *μ*l of dNTPs (10 mM each), 0.5 *μ*l of RNasin (40 U/*μ*l), and 1 *μ*l of M-MLV enzyme were added, and reverse transcription was performed at 37°C for 60 min. Finally, the reaction was terminated at 70°C for 5 min.

Real-Time PCR was done using the Corbett Rotor Gene 6000 Real-Time PCR system (Corbett Research, Australia) and *SYBR Green* Real-Time PCR super master mix (Favorgen, Thailand). The total volume was equal to 20 *μ*l containing 2 *μ*l of cDNA, 10 *μ*M of forward primer (1 *μ*l), 10 *μ*M of reverse primer (1 *μ*l), 10 *μ*l of 2x *SYBR Green* PCR super master mix, and 5.8 *μ*l of dH_2_O. Conditions for PCR included denaturation at 95°C for 3 min, 40 cycles of 10 sec at 95°C, 10 sec at 55-59°C (depending on the primer type), 20 sec at 72°C, and final extension at 72°C for 5 min. The housekeeping gene *β*-actin was used as internal control for protein-coding genes. U67 gene was used as internal control for miR-185 gene. The gene expression ratio was obtained by LinRegPCR software version 2017.0, based on the *ΔΔ*CT method. In order to avoid nonspecific products and primer-dimer products, all PCR products were evaluated by melting curve analysis of the rotor gene. The primer sequences are shown in Table 1.

### 2.8. Cell Culture and Development of PD for the *In Vitro* Model

SHSY-5Y cells obtained from the Institute Pasteur of Iran (IPI) were cultured in DMEM/F12 medium (Invitrogen), supplied with 10% of fetal bovine serum (Invitrogen) and 1% of GlutaMax (Invitrogen), in a humidified atmosphere containing 5% of CO_2_ at 37°C. Rotenone was dissolved in dimethyl sulfoxide (DMSO, final concentration of DMSO was equal to 0.01%). Rotenone (500 nM) was used for 24 h to induce cell damage [24]. In this stage, we wanted to evaluate the changes in the expression level of miR-185, SEPT5, PARK2, and LRRK2 genes in response to rotenone treatment. We also wanted to know if inhibiting each one of the above genes or their products could affect their predicted molecular targets or cellular viability. To address these questions, we designed an experiment on SHSY-5Y cells with eight different treatments. Each experiment was repeated three times.

1. Control positive group (SHSY-5Y cells treated with rotenone for 24 h)
2. Control negative group (SHSY-5Y cells with no treatment)
3. Vehicle group (SHSY-5Y cells treated with rotenone vehicle for 24 h)
4. LRRK2 inhibitor+rotenone group (SHSY-5Y cells pretreated with HG-10-102-01 (50 nM) for 1 h followed by treatment with rotenone for 24 h)
5. LRRK2 inhibitor group (treated with HG-10-102-01 (50 nM))
6. miR-185 inhibitor group (SHSY-5Y cells transfected with a plasmid containing siRNA sequence for inhibiting miR-185 transcript)
7. SEPT5 inhibitor group (SHSY-5Y cells transfected with a plasmid containing siRNA sequence for inhibiting SEPT5 gene transcript)
8. SEPT5 inhibitor+rotenone group (transfected with a plasmid containing siRNA sequence for inhibiting SEPT5 gene transcript, followed by treatment with rotenone for 24 h)

### 2.9. Transfection of SHSY-5Y Cells

Cells at the logarithmic growth phase were plated into 96-well microtiter plates 24 h before transfection, in order to reach 60-80% of confluency. Plasmid DNA (containing SEPT-5 siRNA or miR-185 siRNA) was transiently transfected using FuGENE® reagent (Promega, Madison, WI, USA), according to the manufacturer's protocol. Briefly, 0.1 *μ*l of the FuGENE® 6 Transfection Reagent was mixed into 10 *μ*l of serum-free medium (Opti-MEM® I reduced serum medium) and was incubated for 5 minutes at room temperature. Then, 10 *μ*l of the FuGENE® 6/Opti-MEM® I mixture was added to each well of cells to be transfected and was incubated at 37°C for 48 hours. There was no need to remove serum or change culture conditions or remove the transfection complex. Cells were collected and used in further experiments, 48 hours after transfection.

### 2.10. MTT Assay

Cell viability was measured by the 3-(4,5-dimethylthiazol-2-yl)-2,5-diphenyltetrazolium bromide (MTT) method [27, 28]. As a colorimetric assay, MTT assay can measure the activity of NAD(P)H-dependent cellular oxidoreductase enzyme, so that the enzyme reduces the tetrazolium dye, MTT, into insoluble formazan crystals which has a purple color. Briefly, SHSY-5Y cells were plated at a density of 1 × 10^4^ cells per well in 96-well plates. After exposure to the treatments, 20 *μ*l of MTT (5 mg/ml, Sigma-Aldrich) was added into each well and the cell culture plates were incubated in a humidified incubator at 37°C for 4 hours to allow the formation of purple formazan crystal. Next, 100 *μ*l of the solubilization reagent (0.1 N HCl in anhydrous isopropanol, Sigma-Aldrich) was added into each well, and cell lysates were assessed by spectrophotometric assays, so that the optical density was read at *λ* 570 nm and background was subtracted at 690 nm. Cell viability was shown as a percentage of the value in untreated control cells [24]. All samples were run in triplicate.

### 2.11. Data Analysis

Data were presented by the mean ± SEM of eight rats per group. The dependent *t*-test and one-way ANOVA followed by Tukey's test were used to analyze the statistical significance in two and multiple comparisons, respectively. SPSS software version 23 was used to conduct statistical analyses. *P* values at <0.05 were considered to be significant in all analyses.

## 3. Results

### 3.1. Behavioral Assessment of the Rotenone Model of Parkinson's Disease

**Figure 1:**
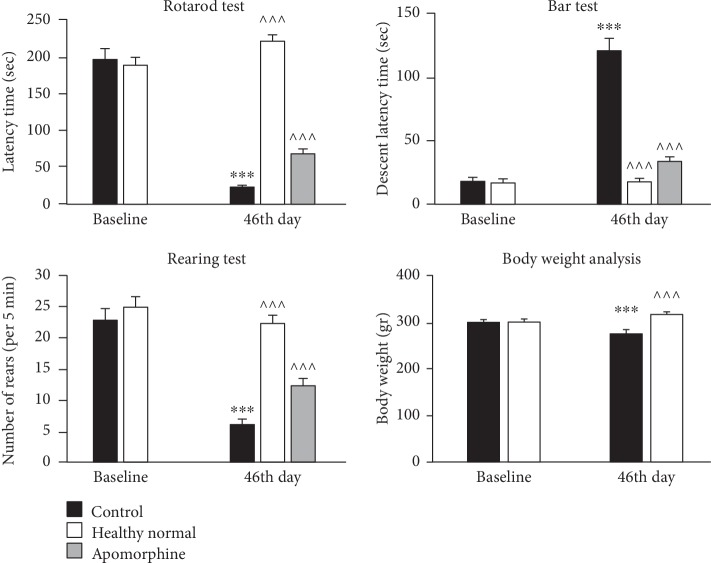
Comparison of the behavioral performance in different experimental groups. The results of the behavioral tests on baseline day and 46th day are shown. Data are presented as mean ± standard error of the mean. *P* value at <0.05 was considered as the significance level. ^∗∗∗^*P* value at <0.001 represents a significant difference compared to the baseline on first day of the experiments. ^^^^^*P* value at <0.001 represents a significant difference in test results of the healthy normal group or the apomorphine-treated group compared to the control group.

The results of three behavioral tests (Rotarod, rearing, and bar tests) showed significant decreases in the muscle strength and balance in the control group compared to both baseline and healthy normal groups (*P* < 0.001) (see Figure 1). Additionally, treatment with apomorphine could reverse the decrease in motor performance on three behavioral tests (rotenone-treated vs. rotenone-treated+apomorphine; (*P* < 0.001) see Figure 1). Furthermore, the body weight analysis showed a moderate weight loss in rotenone-treated groups. Weight difference was statistically significant in the control group compared to baseline and healthy normal groups (*P* < 0.001). However, the decrease in body weight was observed to be only 8.2% compared to the baseline group and 13.2% compared to the healthy normal group.

### 3.2. Gene Expression Analysis of Brain Tissues

**Figure 2:**
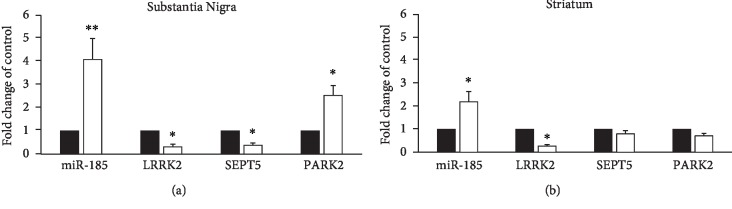
Comparison of the gene expression in the substantia nigra (a) and striatum (b) of the experimental groups. The bands were normalized to beta-actin, and data were expressed as fold change of the control group. Data are presented as mean ± standard error of the mean. *P* values of less than 0.05 were considered as significant. ^∗^*P* values at <0.05 and ^∗∗^*P* values at <0.01 represent a significant difference between the healthy normal group and the control group.

Figure 2(a) shows the results of the gene expression analysis in the transcription level demonstrating that miR-185 (*P* < 0.01) and PARK2 (*P* < 0.05) gene expression significantly decreased in the SN of the control group, compared to the healthy normal group. Furthermore, mRNA of SEPT5 and LRRK2 genes significantly increased in the SN of the control group, compared to the healthy normal group (*P* < 0.05).

In addition, the analysis of the genes' transcripts in the ST of the control group showed a significant decrease in the gene expression of miR-185 (*P* < 0.05) and LRRK2 (*P* < 0.05), compared to the healthy normal group. However, there was no significant difference in the expression of SEPT5 and PARK2 genes between two groups (Figure 2(b)).

### 3.3. Analysis of Gene Expression of SHSY-5Y Cells and Their Viability

**Figure 3:**
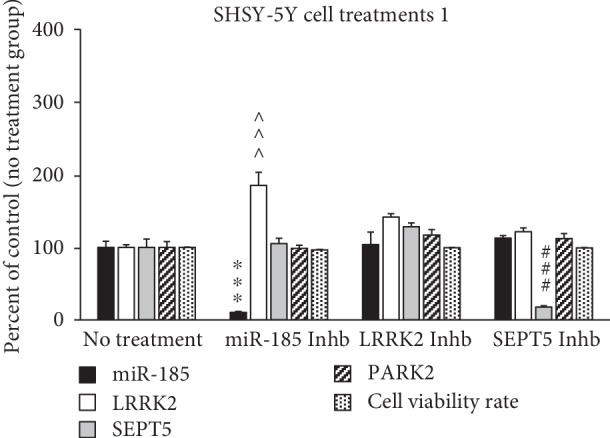
Comparison of the gene expression in SHSY-5Y cell treated by different treatments. The bands were normalized to beta-actin, and data were presented as percentage of the control group. Data are presented as mean ± standard error of the mean. *P* values of less than 0.05 were considered as significant. ^∗∗∗^*P* values at <0.001, ^^^^^*P* values at <0.001, and ^###^*P* < 0.001 represent a significant difference in miR-185, LRRK2, and SEPT5 gene expressions, respectively, between the healthy “no treatment” group and other groups. Abbreviations are as follows: Inhb represents inhibitor; Veh represents vehicle.

**Figure 4:**
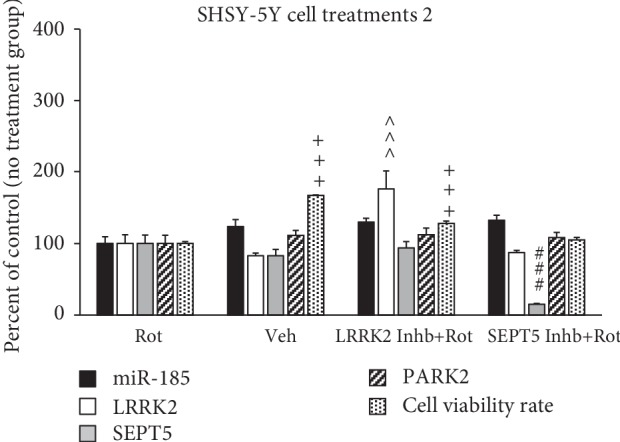
Comparison of the gene expression in SHSY-5Y cell treated by different treatments. The bands were normalized to beta-actin, and data were presented as percentage of the control group. Data are expressed as mean ± standard error of the mean. *P* values of less than 0.05 were considered as significant. ^∗∗∗^*P* values at <0.001, ^^^^^*P* values at <0.001, and ^###^*P* < 0.001 represent a significant difference in miR-185, LRRK2, and SEPT5 gene expressions, respectively, between the Rot (control) group and other groups. ^+++^*P* value at <0.001 represents a significant difference in the cell viability rate between the Rot (Control) group and other groups. Abbreviations are as follows: Rot represents rotenone; Veh represents vehicle; Inhb represents inhibitor.

The results of MTT assay showed that the cell viability rate significantly decreased in SHSY-5Y cells treated by rotenone (control group), compared to the control negative (vehicle) group (*P* < 0.001) (see Figure 3). However, there was no significant difference in the expression of miR-185, LRRK2, SEPT5, and PARK2 genes in rotenone (control group) compared to the control negative (vehicle) group (see Figure 3). The cell viability rate significantly increased due to the inhibition on LRRK2 protein in cells treated by HG-10-102-01 (50 nM) compared to the control group (*P* < 0.001) (see Figure 3). Additionally, the expression of miR-185 gene (*P* < 0.001) decreased and the expression of LRRK2 (*P* < 0.001) gene increased, simultaneously due to the inhibition on miR-185 transcript by its exclusive siRNA (see Figure 4). However, inhibition on SEPT5 transcript by its exclusive siRNA decreased the SEPT5 mRNA level, while could not affect the levels of PARK2, LRRK2, or miR-185 transcripts significantly (see Figure 4).

## 4. Discussion

Results of the movement assessments showed a significant decrease in the performance of the control group indicating that the animal model of PD has been well developed regarding the previous studies [22]. Additionally, apomorphine injection could relieve the signs of the behavioral impairments significantly. Thus, treatment with rotenone could specifically induce dopaminergic cell death in the SN of rats' brain, with the least peripheral effects.

The results of this study showed that the level of miR-185 and PARK2 transcripts significantly decreased in the SN of the control group, while mRNA of SEPT5 and LRRK2 genes significantly increased in the SN of the group, compared to the healthy normal group. Furthermore, the analysis of the genes' transcripts in the ST of the control group showed significant decreases in miR-185 and LRRK2 expressions, compared to the healthy normal group. However, there was no significant difference in the expression of SEPT5 and PARK2 genes in the ST between two groups. These results show the possible role of all the four genes in the development of the PD model and dopaminergic cell death in the SN. However, there was no significant difference in the PARK2 and SEPT5 transcript level in the ST between two groups, which is probably due to the less density of dopaminergic neurons. Additionally, it appears that miR-185 and PARK2 genes play a neuroprotective role in the SN, which lack of them can contribute in dopaminergic cell death and the development of PD. In this regard, Wen et al. reported that the overexpression of miR-185 gene can lead to the inhibition on apoptosis of dopaminergic neurons through regulating the mTOR-dependent autophagy pathway [25]. Furthermore, Elmazoglu et al. reported a decrease in the PARK2 gene expression level in an *in vitro* model of PD induced by rotenone [26]. The increase in the PARK2 gene expression level was also observed in clinical cases of PD or PD models occurred through certain epigenetic processes. For example, certain mutation or decrease in the expression in midnolin (MIDN) gene, observed in almost 10.5% of so-called sporadic PD, can lead to the decrease in PARK2 mRNA [27].

Additionally, previous studies show that the increase in SEPT5 gene expression can lead in dopaminergic cell death through the formation of the Lewy bodies [28, 29]. In current study, the expression of normal LRRK2 increased, while most of other studies only show the increase in the kinase activity of LRRK2 due to the certain mutations [30]. Indeed, those studies neither confirm nor deny the increase in the LRRK2 gene expression level. However, some studies showed the increase in the LRRK2 gene expression level in the microglia present at the SN of the brain in patients with PD occurred due to the inflammation which is an inevitable phenomenon inside the brains of the patients with PD [31, 32]. This reason may be the cause of what was observed about the LRRK2 gene expression level in the current study.

Assessments on SHSY-5Y cells showed no significant difference in all of the four genes between the control group (rotenone-treated group) and the vehicle-treated group. However, the cell viability rate significantly decreased in the control group. These results were not surprising, since we do not expect a perfect similarity in gene expression pattern between *in vitro* and *in vivo* models. The results also showed that inhibition on miR-185 caused by specific siRNA led to the significant increase in the expression level of LRRK2 gene. However, the analysis of gene expression in the SN of rats' brains also showed that there was an inverse relation between miR-185 and LRRK2, but we could not interpret it as a real reliable relation, since we performed no intervention and genetic manipulation on animals. More reliable straightforward results were obtained by assessment of the gene expression level in genetically manipulated SHSY-5Y cells indicating that miR-185 could target LRRK2 mRNA and reduce its expression level. Dweep et al. previously predicted that miR-185 targets LRRK2 mRNA; however, there was no experimental study to confirm the results of their *in silico* study [33]. Current study for the first time showed that LRRK2 could probably be a target for miR-185 transcript. Additionally, the results of this study revealed that inhibition on LRRK2 protein by its specific inhibitor (HG-10-102-01) could prevent rotenone toxicity and lead to the increase in the cell viability rate. In this regard, previous studies showed that the increase in the kinase activity of LRRK2 protein occurred due to the certain mutations (e.g., G2019S) or simply the increase in LRRK2 gene expression can influence its molecular target at least through three pathways: (1) implying the synaptic dysfunction due to the phosphorylation of endothelin A (EndoA), which is a direct target of LRRK2 [34], (2) uncontrolled translation and consequently a bulk increase in protein synthesis through phosphorylation of eukaryotic initiation factor 4E- (eIF4E-) binding protein (4E-BP) and ribosomal protein S15 (RPS15), which induce the formation of the Lewy bodies [35], and (3) deregulation of autophagy processes through influencing Rab7-dependent perinuclear lysosome clustering and lysosomal degradation [36, 37]. Therefore, it appears that the present study found a missing link located in the upstream of the LRRK2-related pathophysiological process. Accordingly, miR-185 gene is likely a noteworthy molecular target, which would be considered in designing upcoming preclinical and clinical therapeutic approaches. It is noteworthy that the increase in LRRK2 gene expression due to the inhibition on miR-185 gene expression could not lead to the decrease in the cell viability rate. However, it is difficult to interpret this finding and further experiments are needed to elaborate this finding, but probably it results from the multifactorial nature of PD, so that only the increase in LRRK2 expression cannot imply enough pressure to induce SHSY-5Y cell death.

The results of the current study showed that inhibition on the expression of SEPT5 gene could neither influence the expression of the other three genes nor prevent the toxicity of rotenone on SHSY-5Y cells. However, it was predictable that SEPT5 gene cannot influence the expression of miR-185, LRRK2, or PARK2, since there was no accordant report or prediction in this regard. Indeed, SEPT5 protein is a molecular target of PARK2, so that a decrease in the expression level of PARK2 can lead to the high cellular amount of SEPT5, contributing in the formation of the Lewy bodies [38]. Thus, we did not manipulate the expression level of PARK2 to evaluate the changes in the expression of SEPT5 gene, since it was predictable based on the previous studies.

## 5. Conclusion

The findings of the study recommended the protective role of miR-185 gene in preventing the development of the PD model, although further preclinical and clinical studies are needed to confirm the findings and to develop novel therapeutic approaches for the treatment of PD based on this finding. SEPT5 gene is also probably involved in the pathophysiological mechanisms of PD. In addition, further studies are recommended to evaluate the probable associations between oxidative stress pathways and miR-185 and SEPT5.

## Figures and Tables

**Table 1:** The primer pairs used for the gene amplifications.

Primers	Sequence (5′ → 3′)	GC%	*T*_*m*_ (°C)	PCR product length (*n*)
SEPT5 (forward)	GCTGAGGAACGCATCAAC	55.6%	56.5°C	167
SEPT5 (reverse)	AACTGCTGGTCTACATAGTC	45.0%	55.2°C	
LRRK2 (forward)	CCTGGATTGCTGGAGATTG	52.6%	57.3°C	175
LRRK2 (reverse)	GAATGGTGAGCCTTGGTTG	52.6%	56.5°C	
PARK2 (forward)	GACGCTCAACTTGGCTACTC	55.0%	57.1°C	131
PARK2 (reverse)	CACTCCTCGGCACCATAC	61.0%	57.6°C	
*β*-act (forward)	CGTGCGTGACATTAAAGAGAAG	45.5%	58.5°C	134
*β*-act (reverse)	CATTGCCGATAGTGATGACC	50.0%	57.6°C	
miR-185 (forward)	Exclusively designed by Bon Yakhteh Company, Tehran, Iran	—	—	—
miR-185 (reverse)	Exclusively designed by Bon Yakhteh Company, Tehran, Iran	—	—	
U67 (forward)	Exclusively designed by Bon Yakhteh Company, Tehran, Iran	—	—	—
U67 (reverse)	Exclusively designed by Bon Yakhteh Company, Tehran, Iran	—	—	

## Data Availability

The data used to support the findings of this study are available from the corresponding author upon request.
